# Preliminary Research on the Possibility of Automating the Identification of Pollen Grains in Melissopalynology Using AI, with Particular Emphasis on Computer Image Analysis Methods

**DOI:** 10.3390/s26072043

**Published:** 2026-03-25

**Authors:** Kacper Litwińczyk, Michał Podralski, Paulina Skorynko, Ewa Malinowska, Zuzanna Czarnota, Beata Bąk, Artur Janowski

**Affiliations:** 1 Department of Geodesy, Institute of Geodesy and Civil Engineering, Faculty of Geoengineering, University of Warmia and Mazury in Olsztyn, 10-719 Olsztyn, Poland; 174172@student.uwm.edu.pl (K.L.); 166411@student.uwm.edu.pl (M.P.); 2 Department of Poultry Science and Apiculture, Faculty of Animal Bioengineering, University of Warmia and Mazury in Olsztyn, Sloneczna 48, 10-957 Olsztyn, Poland; paulina.skorynko@uwm.edu.pl (P.S.); ewamali7@gmail.com (E.M.); zuzanna.czarnota@student.uwm.edu.pl (Z.C.); beata.bak@uwm.edu.pl (B.B.)

**Keywords:** computer image analysis, YOLOv12m, DINOv3, neural networks, deep learning, melissopalinology, bee pollen, identification of type of honey

## Abstract

Melissopalynological analysis is essential for determining the botanical origin of honey, corbicular pollen and bee bread, as well as detecting adulteration. However, it traditionally relies on labor-intensive and subjective manual pollen identification. As a proof-of-concept preceding full honey analysis, this study evaluates artificial intelligence methods for automated pollen grain recognition under controlled conditions. Hazel (*Corylus avellana* L.) and dandelion (*Taraxacum officinale* F.H. Wigg.) were used as model taxa to validate the proposed approach before its application to real varietal honey samples. This study introduces a novel three-stage pipeline that decouples object detection from feature extraction, utilizing YOLOv12m for region-of-interest generation and, for the first time in melissopalynology, DINOv3 ConvNeXt-B for deep feature representation. Microscopic images acquired at 400× magnification yielded 2498 dandelion and 1941 hazel pollen grains. The detector achieved an mAP@0.5 of 0.936 with an F1 score of 0.88, while the classifier reached 98.1% accuracy with good class separability (Silhouette coefficient: 0.407). The primary technical contribution is the systematic optimization of the detection-to-classification interface. Context-aware bounding box expansion (12%) and an optimized IoU-NMS threshold (0.65) significantly improve the stability of morphological feature extraction, as confirmed by ablation studies. Computational cost reporting further supports reproducible, deployment-oriented comparison. The results confirm the feasibility of this AI-based framework as an intermediate step toward automated melissopalynological analysis, with future work focusing on standardized microscopy protocols and expanded pollen databases for varietal honey authentication.

## 1. Introduction

### 1.1. The Melissopalynology

Palynology is a branch of science that studies palynomorphs—that is, all elements appearing in palynological preparations, such as pollen grains, spores, cysts, or diatoms. Among these, pollen grains are the most frequently analyzed object. The term palynology was introduced by Hyde and Williams in 1955 [1]. Palynology provides an effective tool for reconstructing historical vegetation, identifying key plant species beneficial to honey bees, and assessing the impact of climate change on plant populations based on pollen analysis [2]. Melissopalynology is a specialized branch of palynology that focuses on determining the botanical and geographic origin of honey and other bee products such as corbicular pollen or bee bread.

It should be emphasized that the effectiveness of a melissopalynological assessment largely depends on the experience of the analyst. Even minor errors in pollen identification can lead to substantial inaccuracies in later interpretations of the results [3].

Being able to identify the plant sources used by bees in a given area is of significant importance for rational forage management. This capability facilitates proper hive management and broadens opportunities for producing honey with diverse botanical origins [4]. Melissopalynological analyses, in turn, enable the identification of nectar-producing plants, enriching the honey’s value by establishing its botanical and geographic origin [5].

Traditional palynology, which analyses pollen grains and spores, relies on morphological features to identify individual taxa, including shape, polarization, symmetry, apertures, size, and ornamentation of grains. However, small morphological differences between pollen from closely related species hinder rapid and precise classification. Microscopic analysis is time-consuming, costly, and largely dependent on the researcher’s subjective assessment, which can lead to errors reaching as high as about 33%. Despite advances in digital imaging techniques and software that support analysis, the identification process remains largely based on visual evaluation, increasing the risk of errors, especially for less experienced palynologists. Given these limitations, there is a clear need to develop more efficient, objective, and precise methods for pollen grain [6].

Langford et al. [7] show that pollen identification based on morphological features is time-consuming, costly, and prone to subjective errors, especially for similar species. Gonçalves et al. [8] emphasize that traditional pollen identification requires substantial expert knowledge and, due to its time demands, limits its effectiveness in environmental analyses. Balmaki and colleagues (2022) [9] propose applying deep learning for automated pollen identification, which could enhance the precision and efficiency of analyses.

This work makes the following main contributions. First, we propose a modular three-stage pipeline for automated pollen grain recognition that explicitly separates object detection, feature extraction and classification. Unlike traditional monolithic end-to-end models, this decoupling allows for independent optimization of each stage, providing a more robust framework for handling the high morphological variability found in microscopic pollen samples. Second, we introduce a context-aware bounding box expansion strategy together with an experimentally optimized Non-Maximum Suppression and IoU threshold, which jointly improve detection recall, reduce duplicate detections and enhance the separability of learned feature representations in microscopy images. Third, we demonstrate, used for the first time in melissopalynology, self-supervised DINOv3 embeddings provide a highly discriminative representation of pollen morphology, enabling accurate and stable classification using a simple and fast linear classifier. Finally, we present a comprehensive experimental evaluation that includes detection metrics, embedding-space analysis, classifier performance and a detailed computational complexity assessment (FLOPs/Params), setting a new standard for reporting results in automated pollen analysis.

### 1.2. Examples of AI Applications in Melissopalynology

AI has already been employed on multiple occasions within melissopalynology. For example, a study proposed assessing honey authenticity without microscopy. It compared 13 machine learning algorithms including SVM, Random Forest, and ANN to classify seven Spanish honey types based on electrical conductivity and carbohydrate profiles [10]. The necessity of comparing diverse heuristic optimization algorithms is well-documented in other high-demand spatial allocation problems, where methods like Multi-Population-Based Differential Evolution (MDE) have shown superior robustness and speed compared to traditional Particle Swarm or Gray Wolf Optimizers [11]. Beyond chemical properties, neural networks have been used worldwide for automatic pollen grain classification by many independent researchers; for instance, a comparative study of machine learning algorithms including ANN, SVM, and Random Forest demonstrated that segmented and pre-processed hyperspectral images could detect honey adulteration with over 98% accuracy [12]. Convolutional neural networks (CNNs) have established a strong baseline for pollen classification, though their performance varies significantly based on experimental design. For instance, ref. [13] demonstrated that a multi-scale deep learning architecture using a ResNet-50 backbone, combined with a selective deblurring pipeline, could achieve 97.7% accuracy on a large-scale dataset of atmospheric pollen. However, the robustness of such models is highly dependent on how data is split; ref. [14] noted that while a CNN reached 98% accuracy on 83 species during standard splitting experiments, this dropped to 41% in leave-one-out experiments across different flower samples. This disparity highlights a critical gap in the field regarding model generalization to new biological contexts, a challenge we address in this study by using a decoupled pipeline that prioritizes high-quality feature embeddings over simple end-to-end classification. This shift toward machine learning is mirrored in other environmental sciences, such as spatial precipitation modeling, where ANN and ANFIS models have been shown to significantly outperform traditional geostatistical methods in capturing non-linear patterns in complex topographies [15]. Rather than using integrated object detection, some research focuses on the classification of pre-segmented images through specific feature extraction. For instance, Gonçalves et al. established the first annotated dataset for Brazilian Savannah pollen, comparing the performance of three different feature extractors combined with four machine learning classifiers including KNN and SVM. Similar to our solution, YOLOv8 has been used [16] to identify endemic Chilean flora, such as Eucryphia glutinosa. Their model achieved a mean average precision (mAP) of 97.6% for general classes; however, the study noted discrepancies between manual and automatic counts. Our research addresses these discrepancies by moving beyond simple detection to a decoupled YOLOv12m-DINOv3 pipeline, which preserves peripheral morphological details that are often lost in the standard YOLO bounding-box clipping described in their experiments. The effectiveness of the YOLO architecture, particularly when integrated with segmentation algorithms and deep learning for real-time resource detection, has achieved high accuracy rates (e.g., 87.33%) in complex urban monitoring systems like SPARK [17]. The critical role of the detection stage in environmental samples was highlighted by [18], who utilized YOLOv5 to analyze approximately 40 Mediterranean taxa amidst significant background debris. Their findings revealed that approximately 5% of grains remained undetected, primarily due to rare morphologies or poor image quality and that 5% of debris was falsely identified as pollen. This underlines a significant risk of error propagation from the detection to the classification stage, a challenge we mitigate in our pipeline by separating detection from feature extraction and applying optimized IoU-NMS thresholds to improve the filtering of non-pollen artifacts. Comparisons with other neural network architectures have shown that YOLO performs best among them [19,20,21].

The current state of knowledge on automated pollen grain classification, as of the time of writing, appears well explored. The cited studies achieve high classification accuracy.

### 1.3. Research Objectives and Hypotheses

The aim of this study is to evaluate the potential for automating pollen analysis in honey and derived products using artificial intelligence/object detection technologies. The research proposes and verifies a method that will significantly reduce the need for manual identification of individual pollen grains.

Artificial intelligence enables substantial automation of honey pollen analysis, reducing the need for manual identification of each pollen grain.

Implementation of the proposed method will enhance the efficiency of detecting honey samples inconsistent with declared quality levels, resulting in shorter analysis times and reduced human errors.

Automation of pollen analysis will facilitate more effective combating of honey adulteration and improve the reliability of quality control for beekeeping products.

### 1.4. Novelty and Contribution

The novelty of this work does not lie in the introduction of a new neural architecture, but in a reproducible and interface-optimized framework for microscopic pollen recognition. The main contributions are a modular, three-stage pipeline that decouples object detection (YOLOv12m), feature representation (DINOv3), and classification (logistic regression), enabling independent optimization of each stage and reducing error propagation. Furthermore, the detection–classification interface is systematically optimized through context-aware bounding-box expansion and experimentally tuned IoU-NMS settings, with their impact explicitly validated via ablation studies. Finally, this work presents the first application of self-supervised DINOv3 embeddings in melissopalynology, demonstrating strong linear separability of pollen morphology and stable, high classification accuracy achieved using a lightweight linear classifier.

## 2. Materials and Methods

**Figure 1:**
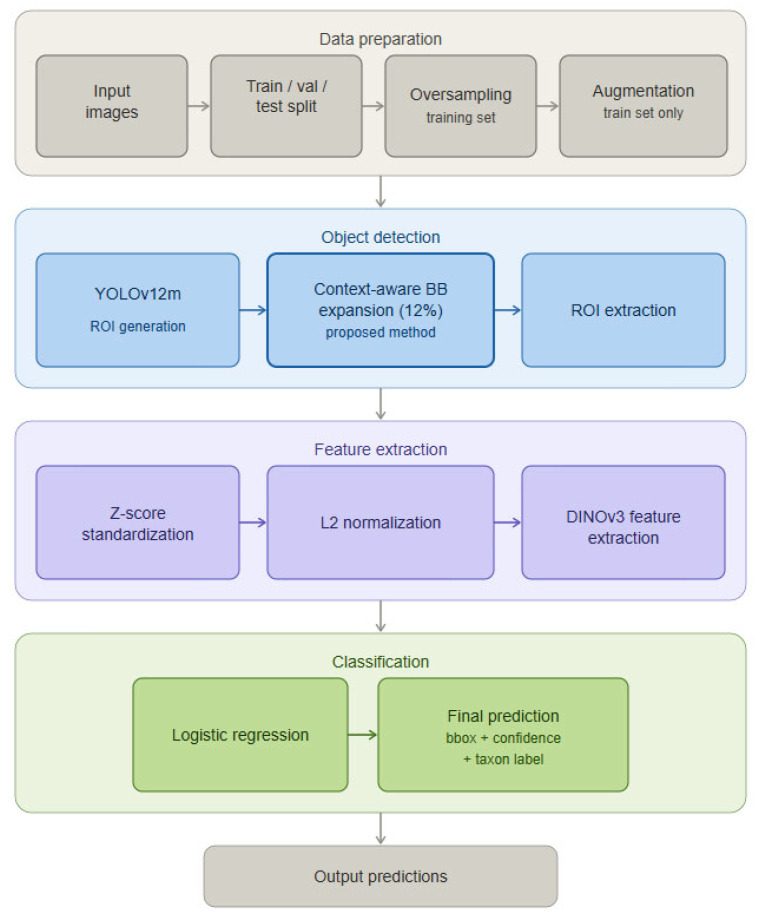
General schematic of data processing in the proposed system.

In the proposed system, raw images are divided into training, validation, and test sets, and then subjected to augmentations (only in the training set). The YOLOv12m model detects objects in the images, from which fragments containing individual pollen grains are cropped. Each crop is analyzed by the DINOv3 model, which generates 1024-dimensional embeddings. These embeddings are classified by logistic regression, producing the final result with class labels and bounding box coordinates (Figure 1).

### 2.1. Sample Acquisition

Pollen from two plant species was used for the study: hazel (*Corylus avellana* L.) and dandelion (*Taraxacum officinale* F.H. Wigg.). These plants represent some of the most important forage sources for honey bees. Based on the predominant pollen originating from a given species (dominant pollen), it is also possible to determine the honey variety.

In order to obtain pollen, flowers of the mentioned species were collected in Olsztyn, Poland during their most intense flowering periods:

hazel—February 2025 r.,

dandelion—May 2025 r.

Selected flowers were selected based on the presence of pollinators, indicating a high pollen content. To prepare microscopic slides, anthers were gently rubbed against the surface of the base glass slide. A drop of water was then applied, and the preparation was covered with a coverslip. The prepared slides were observed under a light microscope.

### 2.2. Images Acquisition

Pollen photographs were taken using an Olympus BX51 optical microscope manufactured by Olympus Company, Tokyo, Japan at 400× magnification. Dedicated cell^F software (Version 1.0) was used for photography, allowing precise image parameter settings and automatic image capture (all images had 2576 × 1932 resolution, color depth 24 bits). The entire process was conducted under laboratory conditions ensuring consistent lighting and proper image sharpness.

**Figure 2:**
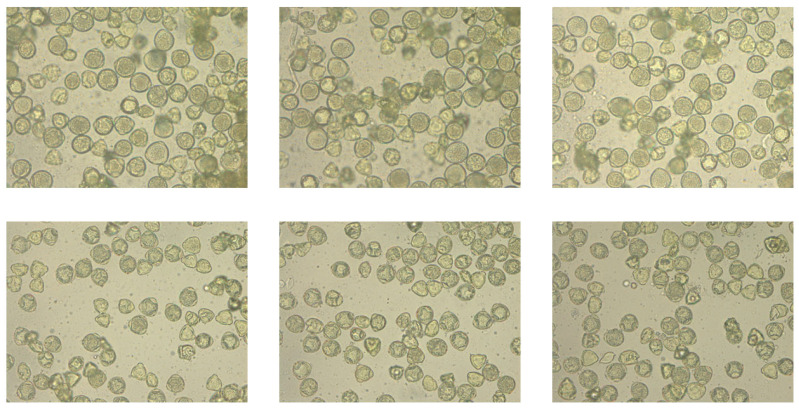
Sample images of hazel pollen grains from dataset used in this article.

**Figure 3:**
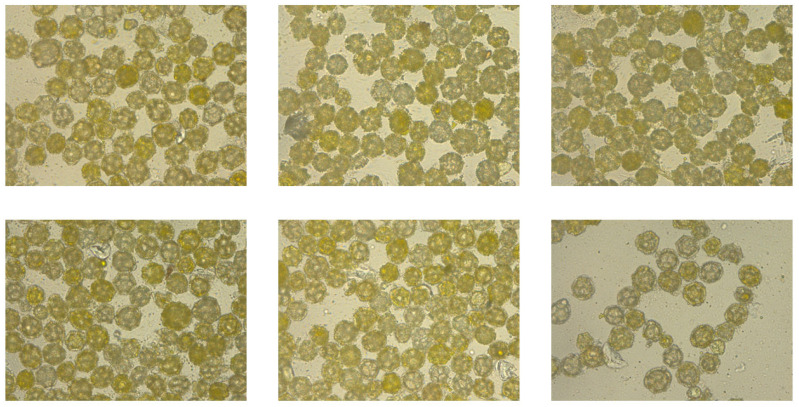
Sample images of dandelion pollen grains from dataset.

A total of 101 images were taken, each depicting a preparation containing only one pollen type. This acquisition method enabled unambiguous pollen identification during subsequent annotation. Results from pollen obtained from flowers (photographs): A total of 51 dandelion pollen photographs and 50 hazel pollen photographs were acquired. The average pollen density per photograph was 49 for dandelion and 39 for hazel (computed as total grains/number of photographs), largely due to differences in grain size between these species. Consequently, a total of 2498 dandelion pollen grains and 1941 hazel pollen grains were photographed (Figure 2 and Figure 3).

### 2.3. Data Annotation

**Figure 4:**
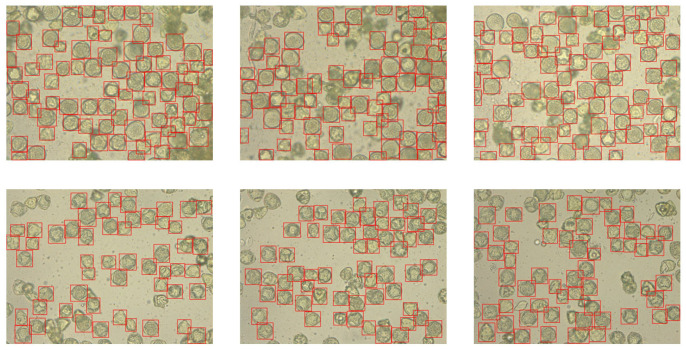
Labeled images of hazel pollen grains from dataset. Labels presented as red frames.

**Figure 5:**
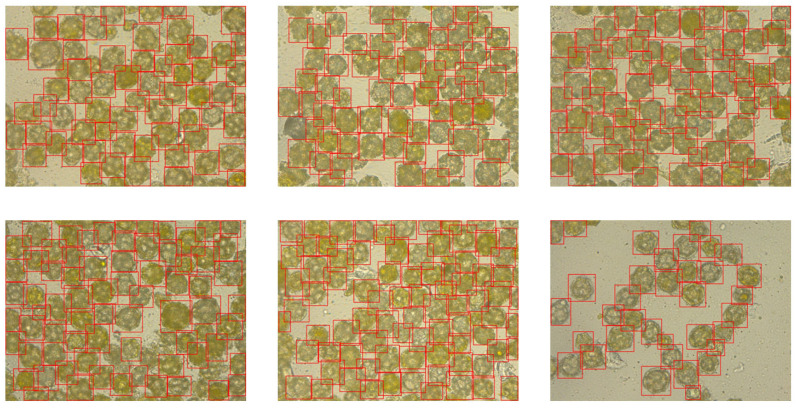
Labeled images of dandelion pollen grains from dataset. Labels presented as red frames.

After acquiring the images, the pollen grains present on them were experienced in pollen work. Thanks to using preparations containing only one pollen type, the annotation process proceeded more efficiently without the need to differentiate each individual pollen grain. The annotation was performed using Label-Studio software (Version 1.19) [22]. To ensure dataset consistency and model reproducibility, annotations were performed according to a standardized protocol. Bounding boxes were defined as ‘tight’ axis-aligned rectangles, encompassing the maximum visible extents of each pollen grain, including the exine ornamentation. Then, 20% of the annotated frames were reviewed to ensure no debris was misclassified as pollen and that ‘cut-off’ grains at the image boundaries were handled consistently. Any discrepancies in classification or box placement were resolved through collaborative review to minimize intra-class variance. Additionally, the bounding boxes generated during annotation were manually verified (Figure 4 and Figure 5) before training using the YoloBBoxChecker program (Version 1.0) [23].

### 2.4. Dataset Preparation

The dataset was first split into training, validation and test subsets before applying any augmentations or oversampling, in order to prevent data leakage. At this stage, the dataset comprised 54 (19 hazel and 35 dandelion) images in the training set, 16 in the validation set and 12 in the test set. The training set consisted of 2780 pollen grains (1720 dandelion and 1060 hazel grains). The validation set included 8 hazel images with 482 pollen grains + 8 dandelion images with 335 pollen grains. The test set consisted of 6 hazel images with 399 pollen grains + 6 dandelion images with 377 pollen grains.

Due to class imbalance in the raw data [24], where dandelion pollen images significantly outnumbered those of hazel, oversampling of the hazel class was applied exclusively in the training set [25]. After oversampling and augmentations, a balanced training dataset was obtained, consisting of 210 hazel images (11,292 pollen grains) and 210 dandelion images (10,320 pollen grains). The validation and test sets were left unchanged in order to prevent data leakage between the training, validation, and test sets. Unchanged duplicates constituted a small portion of the oversamples, with the remainder generated as augmented variants.

**Figure 6:**
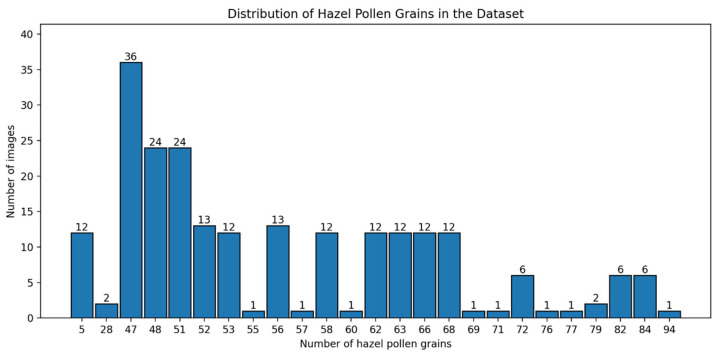
Distribution of pollen grain counts per image for hazel.

**Figure 7:**
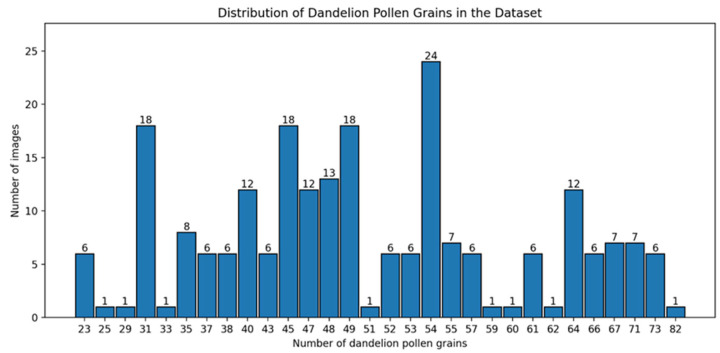
Distribution of pollen grain counts per image for dandelion.

To characterize the structure of the dataset and quantify the variability in pollen grain density across images, we analyzed the distribution of the number of pollen grains per image separately for each class. Figure 6 illustrates the resulting histogram for hazel pollen and Figure 7 illustrates the histogram for the dandelion images. This analysis was performed to assess differences in spatial density and sample heterogeneity, which are known to influence detection difficulty in microscopic images.

**Figure 8:**
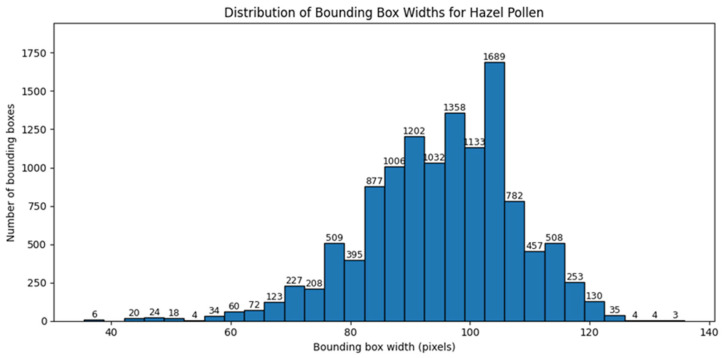
Distribution of bounding box width in pixels for hazel pollen grains.

**Figure 9:**
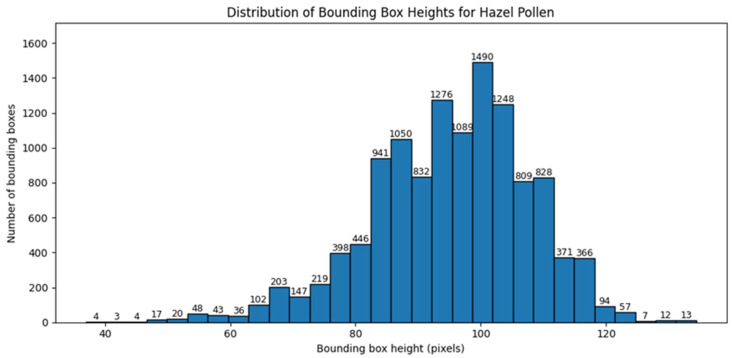
Distribution of bounding box height in pixels for hazel pollen grains.

**Figure 10:**
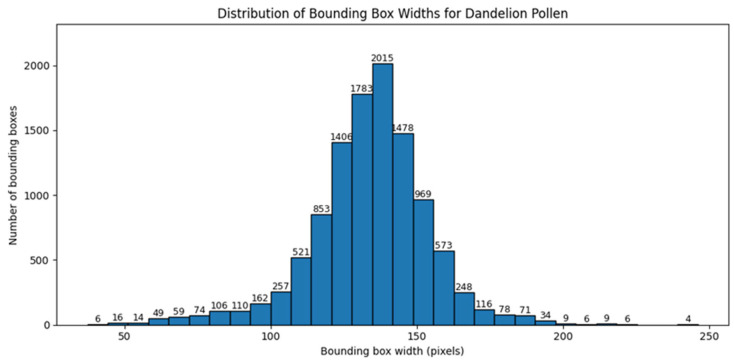
Distribution of bounding box width in pixels for dandelion pollen grains.

**Figure 11:**
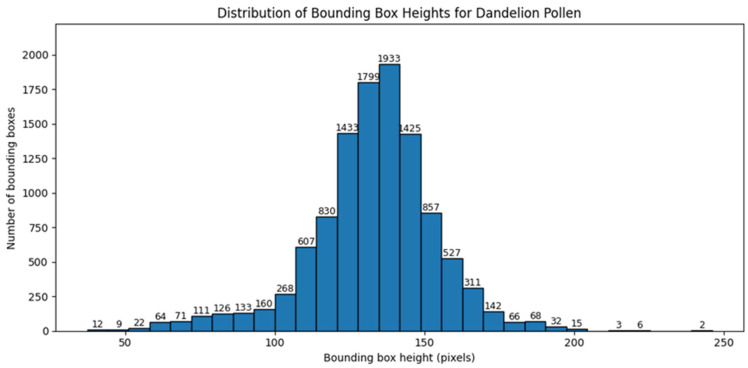
Distribution of bounding box height in pixels for dandelion pollen grains.

Similar analysis was performed for bounding box dimensions expressed in pixels. Figure 8 and Figure 9 present the distributions of bounding box width and height for hazel pollen. Figure 10 and Figure 11 present the distributions of bounding box width and height for dandelion pollen.

**Figure 12:**
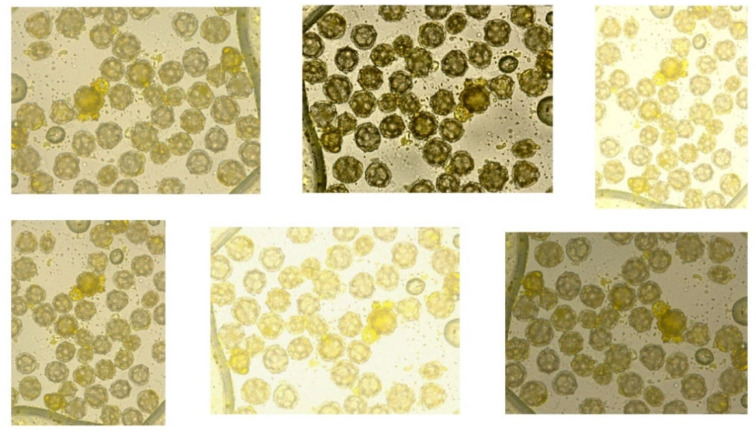
Original image of dandelion pollen grains (**upper left** corner) with its augmentations. In total, after data augmentation and oversampling, we obtained 448 pollen images, which corresponds to 23,205 pollen grains.

Augmentations [26] were applied using the Albumentations library [27] while preserving bounding box consistency. For each training image, 5 variants were generated. Transformations were performed that avoid introducing black bars or letterboxing and do not alter object geometry, except for 90-degree rotations. Specifically, geometric augmentations included horizontal and vertical flips (each with probability *p* = 0.5) and rotations by multiples of 90° (*p* = 0.5). Rotations were limited to 90°/180°/270° angles [28]. Arbitrary-angle rotations require rectification using padding or significant cropping, which generates artificial areas (so-called black bars) and sharp edges absent in the source data. Such artifacts are easily detectable by detection networks and can become undesired learned signals, leading to domain shift between the training set and validation or test sets. Using 90° multiples preserves image dimensions without padding, thereby avoiding artifact introduction, maintaining YOLO-format annotation consistency, and stabilizing the training and evaluation process. Photometric adjustments and degradations such as noise, blur, compression, or scale reduction reflect real-world microscopic data acquisition conditions, ensuring variations in lighting, sharpness, and image quality. In practice, these included random brightness and contrast adjustment (±20%, *p* = 0.7), adaptive histogram equalization (CLAHE, clip limit 2–4, *p* = 0.3), gamma correction (γ ∈ [0.8, 1.2], *p* = 0.4), multiplicative noise (multiplier ∈ [0.9, 1.1], *p* = 0.3), and ISO noise (*p* = 0.3). Additionally, one operation from a set of blur or sharpening transformations (Gaussian blur, motion blur, defocus blur, or sharpening) was applied with probability *p* = 0.6. Mild downscaling (scale factor 0.75–0.95, *p* = 0.2) and JPEG compression (quality range 60–95, *p* = 0.2) were also employed. Absence of strong geometric transformations preserved the actual pollen morphology, such as shape characteristics and proportions. Below is a complete set of augmentations for one original image (Figure 12).

### 2.5. Pipeline Overwiew

**Figure 13:**
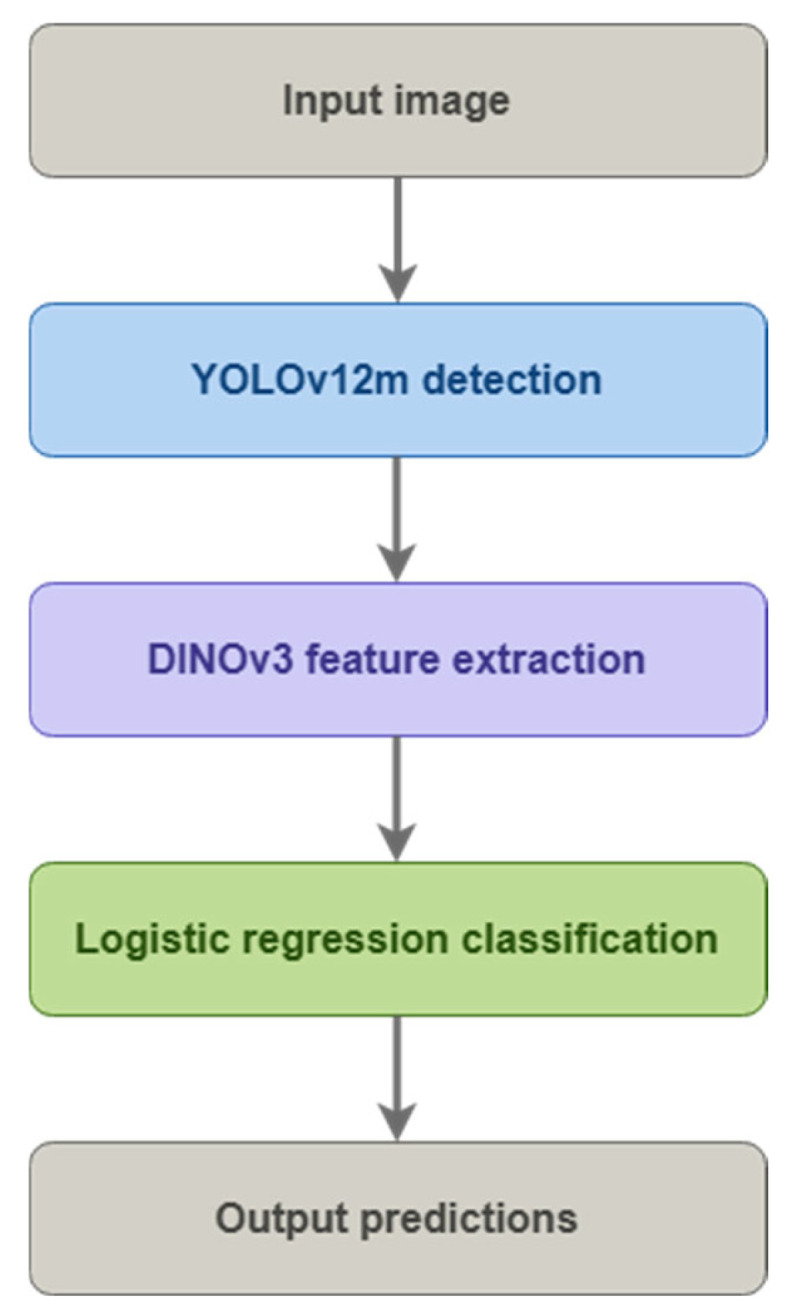
Pipeline consisting of three sequential stages: Object detection (YOLOv12m), Feature extraction and embeddings generation (DINOv3), Embeddings classification (logistic regression).

A detection–classification system was designed, in which the YOLOv12m model serves as the object detector, the modern DINOv3 ConvNeXt-B model [29,30] developed by researchers at Meta acts as the feature extractor, trained on the LV1689M dataset, and logistic regression functions act as the final classifier operating on embeddings generated by DINOv3. Each step of the pipeline is described in the following chapters (Figure 13).

### 2.6. Stage 1: Detection (YOLOv12m)

YOLOv12m was selected as the object detector due to its favorable trade-off between detection accuracy and computational complexity. According to the official YOLOv12 benchmarks, the m variant provides a substantial improvement in detection performance over smaller variants (mAP 0.5–0.95 ≈ 52.5) while maintaining significantly lower parameter count and inference cost than the l and x models [31]. The suitability of YOLO-based detectors for this task is further supported by previous studies on dense small-object detection. For instance, Chen et al. 2024 [32] demonstrated that optimized YOLO architectures significantly improve detection accuracy for small and densely packed objects. Bounding box filtering was performed using the Intersection over Union Non-Maximum Suppression (IoU-NMS) Equation (1) algorithm, whose purpose is to remove duplicate detections.

(1) $$IoU=\frac{\left|A\cap B\right|}{\left|A\cup B\right|}$$

A—Area of first bounding box

B—Area of second bounding box

The NMS threshold was set to *IoU* = 0.65, which in practice allows only those bounding boxes representing objects with the highest confidence to remain.

### 2.7. Stage 2: DINOv3 Embeddings + L2 Normalization

For feature extraction, DINOv3 with a ConvNeXt-Base backbone was chosen due to its strong performance in self-supervised representation learning and its ability to produce highly transferable embeddings without task-specific fine-tuning [33]. Prior work has shown that DINOv3 representations exhibit stronger linear separability and robustness across downstream tasks. This model generates 1024-dimensional embeddings for each sample Equation (2), i.e., compact feature vectors describing the object’s structure, texture, and appearance.

(2) $$f_{i}={\left[\;f_{i1},\;f_{i2},\;\ldots ,\;f_{i,1024}\right]}^{T}\;\in \;R^{1024}\;$$

where f_ik_ denotes the k-th value of the embedding vector.

Embedding can be treated as a point in feature space, where objects with similar structures are close to each other and different objects are far apart.

To ensure comparability of vectors and reduce the impact of their scale, embeddings are subjected to L2 normalization Equation (3), which means transformation:

(3) $$\hat{f_{i}}\;=\;\frac{f_{i}}{||f_{i}|{|}_{2}}$$

where the Euclidean Equation (4) norm is given by:

(4) $${\left||f_{i}|\right|}_{2}\;=\;\sqrt{\sum_{k=1}^{1024}{f_{ik}}^{2}}$$

where:

${\left||f_{i}|\right|}_{2}$—Euclidean norm [34]

$f_{ik}$—*k*-th embedding component

$\hat{f_{i}}$—standardized feature vector

$f_{i}$—unprocessed embedding of the *i*-th sample

After Euclidean normalization, each embedding satisfies ${\left||{\hat{f}}_{i}|\right|}_{2}\;$= 1 meaning it lies on the surface of a hypersphere in R^1024^ space.

After applying L2 normalization, the scalar product of two embeddings corresponds to the cosine similarity Equation (5):

(5) $${\hat{f_{i}}\;}^{T}\hat{f_{j}}\;=\;cos\;∠\;(f_{i},f_{j})\;$$

where:

$\hat{f_{i}}$—embedding of *i*-th sample

$\hat{f_{j}\;}$—embedding of *j*-th sample

This approach achieves geometric separation, ensures reliability in pattern matching, and provides noise reduction.

The value of the expression $\hat{f}$_i_^T^$\hat{f}$_j_ ∈ [−1, 1] is interpreted as a similarity measure between two samples; the closer the value is to 1, the more similar their representations in the feature space.

In practice, L2 normalization facilitates the geometric separation of classes, stabilizes pattern matching, and mitigates the impact of noise and illumination variations. In the subsequent stages of the system, the embeddings $\hat{f}$_i_ serve as input to the classifier.

### 2.8. Stage 3: Logistic Regression Classifier

Logistic regression was selected as the final classification model to deliberately evaluate the discriminative quality of the learned embeddings rather than classifier complexity. As a linear model, logistic regression provides an interpretable decision boundary and is commonly used as a linear probing classifier to evaluate the linear separability of representation spaces learned by deep or self-supervised feature extractors [35]. This design choice ensures that the reported classification performance primarily reflects the quality of the feature representations extracted by DINOv3, while minimizing the risk of overfitting associated with more complex classifiers. The third stage consists of training a multiclass logistic regression model. Logistic regression was chosen as the final classifier due to its simplicity, interpretability, and strong performance in linearly separable embedding spaces, allowing the quality of the learned representations to be evaluated without introducing additional model capacity. Prior to feeding the data into the logistic regression, z-score standardization Equation (6) of the embedding components was applied, which stabilizes the optimization, unifies the feature scales, and significantly improves the numerical conditioning of the logistic regression.

(6) $$x_{ij}=\frac{\hat{f_{ij}}-\mu_{j}}{\sigma_{j}}$$

where:

$x_{ij}$—standardized j-th component of i-th sample

$\hat{f_{ij}}$—the j-th component after L2 normalization

$\mu_{j}$—the mean value of the j-th feature component across the training set

$\sigma_{j}$—the standard deviation of the j-th feature component across the training set.

This results in x_i_ = [x_i1_, …, x_i, 1024_]^T^.

Although subsequent z-score standardization removes the unit norm property, prior L2 normalization preserves the angular structure of the embedding space. Z-score standardization then equalizes variance across dimensions and improves the numerical conditioning of the classifier without reintroducing scale-dependent artifacts

Standardization unifies the scales of individual features and improves the numerical properties of the learning process.

The vector $x_{i}\in R^{1024}$ is fed into a multiclass logistic regression. The model estimates the class probabilities using the softmax function Equation (7).

(7) $$P(y=j\;|\;x_{i})=\frac{exp({w_{j}}^{T}x_{i}+b_{j})}{\sum_{k=1}^{K}exp(w_{k}^{T}x_{i}+b_{k})},\;j=1,\;...\;,K$$

where:

$w_{j}\;\in R^{1024}$—weight of the j-th class

$b_{j}$—the bias associated with the j-th class

K—number of classes

Combining L2 normalization with subsequent z-score standardization ensures that the classifier primarily operates on angular relationships between embeddings, while minimizing the influence of absolute scale, calibration, and signal intensity. Consequently, the classifier’s decision relies mainly on the structure and direction of the feature vector, rather than artifacts from signal amplitude.

To justify the selection of a lightweight linear classifier, a comparative evaluation was conducted against two alternative baselines on the same ROI crops: a shallow feature-based pipeline employing Histogram of Oriented Gradients (HOG) descriptors with an SVM classifier, and a conventional deep learning baseline based on an ImageNet-pretrained ResNet model fine-tuned for binary classification. For all compared methods, ROI crops were generated using the identical bounding-box extraction procedure and padding policy adopted in the proposed pipeline, ensuring that any performance differences are attributable to the classifier and feature representation rather than to discrepancies in ROI generation.

### 2.9. Training Details

The project employed the YOLOv12m model, as it represents the most balanced variant in the YOLO12 family: it offers high detection accuracy (mAPval 52.5), reasonable computational cost, and training stability, while retaining the advantages of the new attention-centric architecture. In the context of detecting numerous small objects in microscopic images, this model provides the best compromise between accuracy, speed, and hardware requirements.

Training of the YOLOv12m model was conducted in the Paperspace cloud environment on a Free-A4000 machine (NVIDIA A4000 GPU 16 GB, 45 GB RAM, 8 vCPU). The model was trained for a maximum of 80 epochs, but the process was automatically stopped around epoch 65 using early stopping, which monitored the lack of improvement in the validation metric. This mechanism prevented overfitting and shortened computation time.

Direct down sampling of the original images to the network input resolution was deliberately avoided to preserve fine-scale structures critical for microscopic object detection and to maintain a safety margin for very small objects close to the lower detectability limit. Early aggressive resizing would irreversibly propagate interpolation errors throughout the entire training process, potentially degrading recall. Retaining higher intermediate resolution prior to the framework-level letterbox resizing also ensured greater flexibility of data augmentation strategies, as operations such as random scaling or mosaic are known to be less effective on already down sampled inputs. Although this design choice resulted in a reduced batch size and increased preprocessing overhead, it provided a more conservative and methodologically robust training setup, reducing the risk of information loss that could otherwise be questioned in the case of aggressive early down sampling

Despite the small batch size of size 2, the model exhibited stable convergence thanks to GPU acceleration and optimizations in the Ultralytics implementation.

To avoid information leakage between the training, validation, and test sets, data splitting was performed at the level of entire microscopic images: each image along with its corresponding YOLO annotations was assigned exclusively to one of the subsets (train/val/test). Oversampling of the hazel class and all augmentations (e.g., mosaic, random shifts and scalings, photometry) were applied only to the training set, while the validation and test sets remained not augmented.

During training, the detector saw only the training set, while threshold selection (early stopping, patience: 15) and metric monitoring were performed on an independent validation set (val: true, split: val). The test set was used only once, namely for the final model evaluation after training completion. This ensured that test set information did not influence the training process or hyperparameter tuning.

The initial learning rate was set to lr = 0.01, then linearly decreased during training to a final value of 0.0001. Three warmup epochs were applied, during which the learning rate was smoothly increased from near zero to the starting value, stabilizing the initial training phase. After warmup, the learning rate was linearly reduced until the end of training. Stochastic gradient descent (SGD) with momentum was used for weight optimization, with momentum = 0.944 and L2 regularization with weight decay = 0.0005×. Training was conducted in mixed-precision mode, which accelerated GPU computations while maintaining numerical quality. To ensure reproducible results, a fixed random seed and deterministic mode were used.

For feature extraction, DINOv3 with ConvNeXt-B architecture was used, trained on the large LV-1689M image dataset, as it belongs to the new generation of self-supervised learning methods that enable the model to build stable, semantic image representations without the need for manual labeling.

As a result, the embeddings generated by the DINOv3 architecture exhibit significant resilience to variations in lighting, contrast, and noise, while effectively capturing the underlying structural patterns of the objects. Furthermore, this robustness facilitates superior transferability to small, specialized datasets, such as the microscopic preparations used in this study.

This is crucial when working with microscopy, where annotations are typically scarce. ConvNeXt-B represents a family of improved convolutional networks inspired by Transformers, but optimized for training stability, computational efficiency and greater model depth and capacity.

Unlike Transformers, ConvNeXt excels with high-resolution images typical of microscopic ones, without a drastic increase in memory costs. This makes it an ideal feature extractor for systems where both representation quality and computational efficiency matter.

The model was pre-trained on the extensive LV-1689M dataset, which encompasses hundreds of millions of diverse images across varied structures, textures, and contexts. Leveraging this foundational training allows the model to map a highly rich feature space, facilitating robust generalization even to specialized data with significantly different distributions, such as microscopic pollen imagery. Furthermore, this approach bypasses the necessity of training from scratch on small datasets, effectively mitigating the risk of overfitting and ensuring more stable classification performance.

For classifying embeddings generated by the DINOv3 model, multiclass logistic regression was applied, as L2-normalized embeddings form well-separated clusters in the feature space, making the classification task largely linear and eliminating the need for more complex classifiers. Logistic regression is numerically stable, resistant to overfitting on small training sets, and fully leverages the geometric properties of embeddings (angular distance). At the same time, it ensures very high classification effectiveness, making it a natural and optimal choice in this pipeline stage.

### 2.10. Evaluation Metrics

Computational complexity and inference time:

The computational cost of the proposed pipeline was evaluated using the final trained models. Inference-time measurements were conducted on a local workstation equipped with an NVIDIA GPU GeForce RTX 4050 and 32 GB of Random Access Memory. The average inference times for individual pipeline components are summarized in Table 1.

In addition to inference time, we report model size and computational cost. The YOLOv12m detector contains approximately 20.1 M parameters and requires 67.1 GFLOPs per inference at an input resolution of 1280 × 1280, as reported by the training framework. The average training time was 1.5 h for 66 epochs with early stopping.

The DINOv3 feature extractor was used in frozen mode and its backbone contains 89 M parameters. The logistic regression classifier introduces a negligible number of parameters and computational overhead

**Figure 14:**
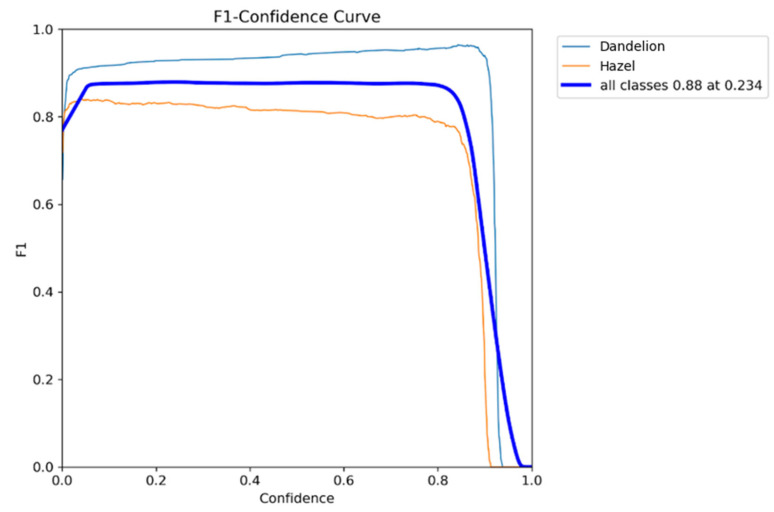
The F1–Confidence curve illustrates the impact of the confidence threshold on detection quality.

The F1–Confidence curve (Figure 14) shows the relationship between the YOLO detector’s confidence threshold value and prediction quality measured by the F1 metric [36]. This curve enables determination of the optimal decision threshold, balancing detection precision and sensitivity [37].

The analysis reveals a broad stability plateau across a wide range of confidence thresholds. For both dandelion and hazel classes, the F1 score remains high and nearly constant for confidence values between approximately 0.05 and 0.75, indicating strong robustness of the YOLO detector to threshold selection and good object separability with low false-alarm rate.

The aggregated curve reaches its global maximum at a confidence threshold of 0.234, achieving an F1 score of 0.88. This value represents the optimal balance between precision and recall for both classes jointly. Lower thresholds increase the number of detections at the cost of additional false positives, whereas higher reduce recall by rejecting correct detections. The selected values therefore maximize overall detection quality.

Class-wide analysis shows consistently higher F1 values for dandelion across the entire confidence range, reflecting better visual separability and more distinctive morphological features. In contrast, the hazel class exhibits slightly lower F1 scores, likely due to greater intra-class variability, partial interspecies feature overlap, and more challenging visual conditions. Nevertheless, the hazel curve remains flat and stable, indicating reliable performance without abrupt quality degradation.

A sharp decline in F1 is observed for confidence thresholds above approximately 0.85 for both classes. This behavior is attributed to excessive recall penalization and rejection of valid detections with moderately lower confidence scores. Consequently, very high confidence values are suboptimal for dense microscopy scenarios involving small objects such as pollen grains.

The analysis additionally applied a threshold optimization method based on minimizing the distance of curve points (confidence, F1) Equation (8) to the ideal point (1,1). This methodology finds a compromise between high prediction quality (F1) and high model confidence.

For each threshold value, the Euclidean distance was calculated:

(8) $$d=\sqrt{{\left(1-confidence\right)}^{2}+{\left(1-F_{1}\right)}^{2}}$$

The point on the curve with the smallest d indicates the optimal balance between prediction confidence and detection quality. Results showed that the minimum distance point falls almost exactly at confidence threshold = 0.23–0.24, practically coinciding with the value determined from the maximum F1 (0.234).

**Figure 15:**
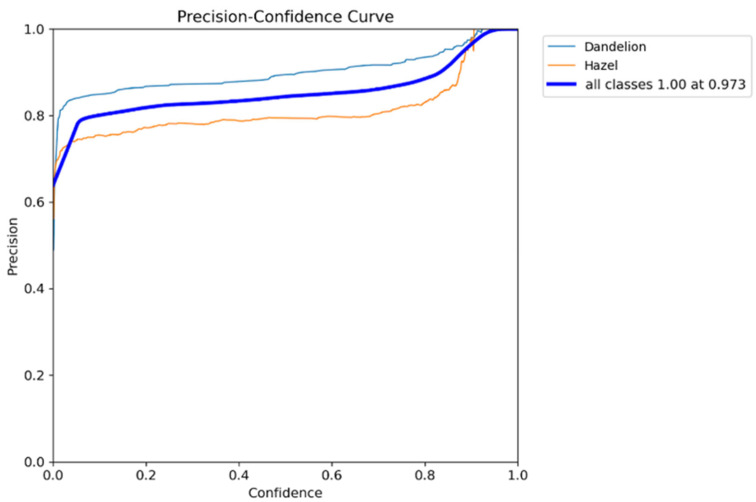
The Precision–Confidence curve for the YOLOv12m detector.

The Precision–Confidence curve (Figure 15) illustrates the relationship between the detector’s confidence threshold and precision. For analysis consistency, the confidence threshold of 0.234 was used, previously determined as optimal based on F1 value (F1 maximum = 0.88 on the F1–Confidence curve).

Read from the Precision–Confidence curve, precision at this threshold is approximately 0.92 for dandelion class and about 0.85 for hazel class, while the aggregated curve (all classes) reaches around 0.88.

This choice of confidence = 0.234 ensures not only maximum F1 metric but also maintains high detection precision for both classes, representing a compromise between minimizing false alarms (high precision) and sustaining high object detectability (high recall), confirming its validity as the model’s operational threshold.

**Figure 16:**
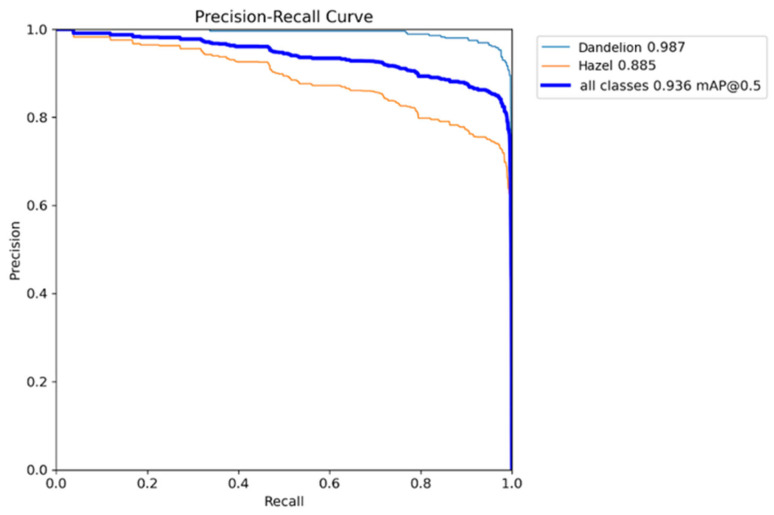
The Precision–Recall curve of the YOLOv12m detector for dandelion and hazel classes.

The Precision–Recall curve [38] (Figure 16) shows the relationship between model precision and sensitivity for both pollen classes, along with the averaged result. Across a wide range of recall values, both classes maintain high precision, indicating that the detector rarely generates false detections when sensitivity is gradually increased.

For the dandelion class, average precision (AP) [39] is 0.987, signaling excellent class separation—even at high recall values, precision stays near 1.0, with quality drop only at extreme sensitivity. The hazel class proves more challenging, with lower average precision of 0.885 and faster precision decline as recall rises, pointing to more false detections in borderline cases.

The aggregated curve (“all classes”) achieves mean average precision for IoU = 0.936, a very strong result given microscopic images, limited samples, and varied pollen morphology. This means the model delivers high object detectability and low false alarms across nearly the full usable sensitivity range.

**Figure 17:**
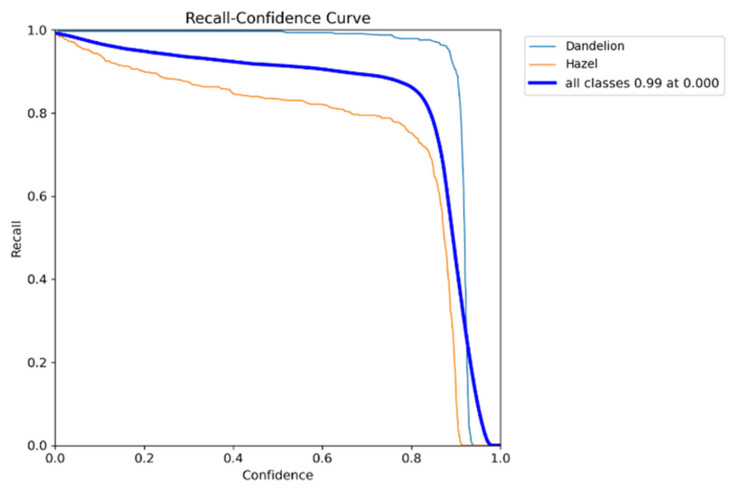
The Recall–Confidence curve of the YOLOv12m detector for dandelion and hazel classes.

Analysis of the Recall–Confidence (Figure 17) curve [40] shows that the model maintains high sensitivity across a wide range of confidence thresholds. For the dandelion class, the detection rate stays near 100% up to a threshold of about 0.90, meaning detections remain stable even with aggressive rejection of low-confidence bounding boxes. For the hazel class, recall drops earlier, around 0.60 to 0.80 for confidence thresholds > 0.60, confirming the greater difficulty of this class and higher model uncertainty regarding these objects.

**Figure 18:**
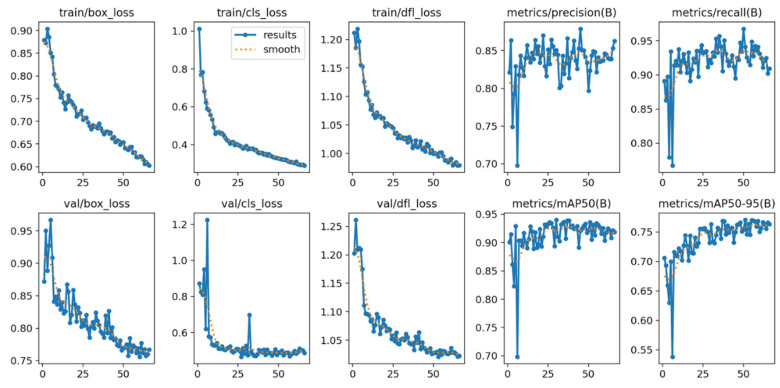
The training process curve for the YOLOv12m detector shows training and validation losses along with quality metrics (precision, recall, mAP).

The charts (Figure 18) depict the training process of the YOLOv12m model, including loss function values (box_loss, cls_loss, dfl_loss) for both training and validation sets, as well as key detection quality metrics: precision, recall, mAP@50, and mAP@50–95.

Loss Convergence:

Training losses (train/box_loss, train/cls_loss, train/dfl_loss) show a monotonic, smoothed decline across epochs, indicating proper model optimization and absence of gradient instability. This pattern reflects a well-chosen learning rate and stable optimizer performance. Validation losses (val/box_loss, val/cls_loss, val/dfl_loss) follow a similar trend with greater variance, natural for small validation sets and high object density. Key observations include no systematic validation loss increase, no train–validation divergence signaling overfitting absence, and stabilization after ~25–30 epochs. Training stopped around epoch 65 per early stopping (patience = 15) due to no further validation metric improvement.

Detection Metrics:

Precision gradually rises and stabilizes at ~0.85–0.90 for aggregated classes, signifying low false positives (FP), crucial for dense microscopic data. Recall exhibits a rising trend, stabilizing at 0.90–0.95, confirming high true-positive capture for numerous small pollen grains. Both metrics improve together, maintaining balance without trade-offs. mAP Metrics:

mAP@50 [41] stabilizes above 0.90, denoting excellent detection at IoU = 0.5 tolerance. mAP@50–95 reaches 0.75–0.80, impressive for small-object detection, limited data, and morphological diversity; this stricter multi-threshold metric accurately reflects overall model quality.

**Figure 19:**
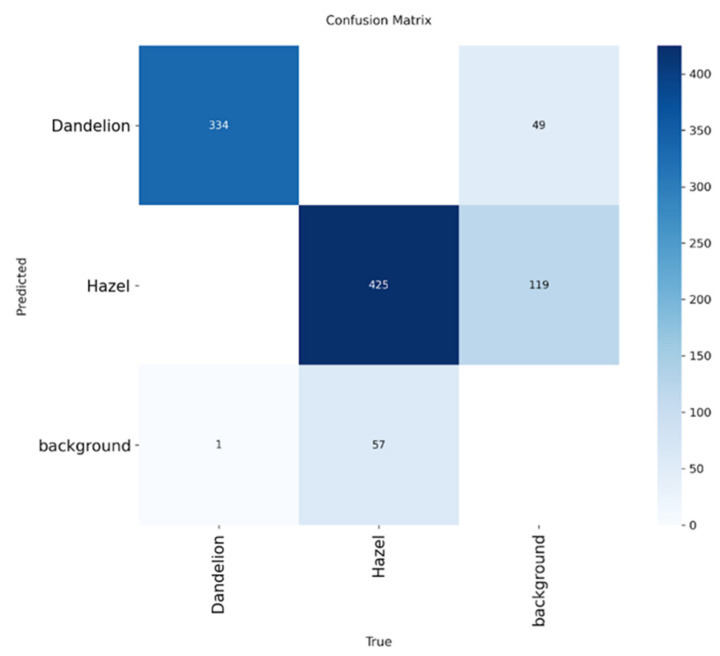
The YOLOv12m detector’s confusion matrix for three classes: dandelion, hazel, and background.

The confusion matrix [42] (Figure 19) summarizing the detection and classification performance of the proposed pipeline for three categories: dandelion, hazel and background (false detections). Each matrix entry represents the number of predictions assigned to a given class with respect to the ground truth.

For the dandelion class, 334 instances were correctly classified, while 49 detections were rejected as background. Importantly, no dandelion samples were misclassified as hazel. The observed errors correspond exclusively to uncertain or low-quality detections that were conservatively filtered out as background rather than incorrectly assigned to another pollen class.

Similarly, the hazel class achieved 425 correct classifications, with 119 instances misclassified as background and no cases confused with dandelion. The absence of cross-species confusion confirms that the model reliably distinguishes between the two pollen taxa. The higher number of background rejections for hazel reflects its greater morphological variability and more challenging visual characteristics, such as partial occlusions, blur or overlap between grains.

For the background category, only one false positive was assigned to dandelion, whereas 57 background regions were misclassified as hazel. This asymmetry indicates that hazel-like structures are more prone to false detections, which is consistent with the lower precision observed for the hazel class in the Precision–Recall and F1–Confidence curves.

Overall, the confusion matrix demonstrates that the proposed pipeline effectively eliminates inter-class confusion between pollen species. Most errors arise from conservative background rejection rather than incorrect species assignment, supporting the robustness of the detection–classification strategy and its suitability for high-confidence pollen identification.

**Figure 20:**
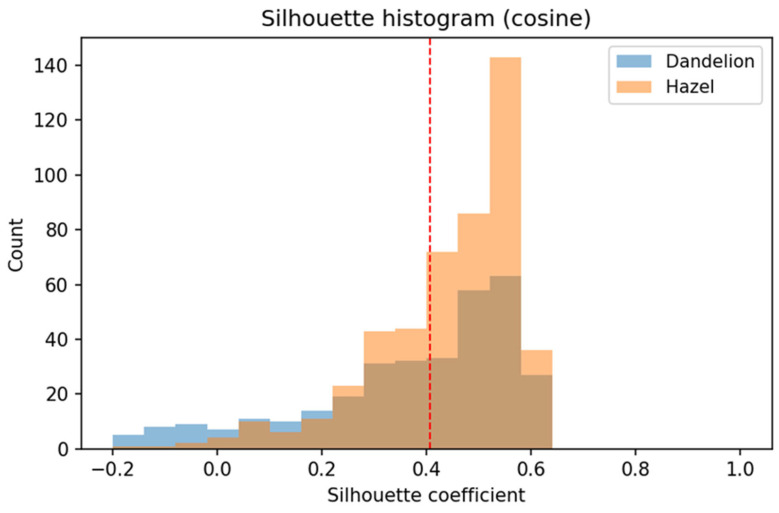
Histogram of Silhouette values for DINOv3 embeddings (cosine measure). The red line indicates the average silhouette coefficient (avg = 0.407).

Distribution of the Silhouette coefficient (Figure 20) computed in the DINOv3 embedding space using cosine distance. This metric quantifies class separability by jointly considering intra-class compactness and inter-class separation, with values closer to 1 indicating well-separated clusters and values near or below 0 indicating overlap.

The average Silhouette value is approximately 0.40, which indicates a clear and stable separation between the two pollen classes in the learned embedding space.

The hazel embeddings exhibit a pronounced shift toward higher Silhouette values, predominantly in the range of 0.35–0.65, reflecting high intra-class cohesion and minimal overlap with the dandelion cluster. This suggests that DINOv3 effectively captures discriminative morphological characteristics of hazel pollen grains.

In contrast, dandelion embeddings show a broader distribution spanning from slightly negative values up to approximately 0.6. This wider spread indicates greater intra-class variability and the presence of borderline samples, which is consistent with the higher morphological diversity observed in dandelion pollen micrographs. The limited occurrence of negative Silhouette values is expected in a two-class setting and does not indicate systematic misclustering.

Overall, the Silhouette analysis confirms that the DINOv3 embeddings form a well-structured and discriminative feature space, providing favorable conditions for downstream linear classification. Together with the high classification accuracy achieved by the logistic regression classifier, these results validate the quality and robustness of the feature representations.

**Figure 21:**
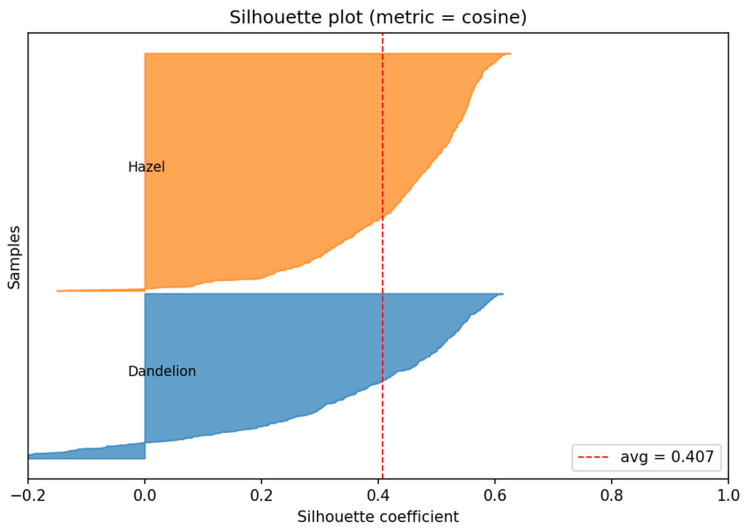
Silhouette plot for DINOv3 embeddings.

Silhouette coefficient values (Figure 21) for all samples from both classes are shown below, computed in the DINOv3 embedding space using cosine distance. Silhouette measures cluster separation degree, with values near 1.0 indicating well-separated clusters, values around 0 signifying overlap, and negative values evidencing misassigned samples.

The average Silhouette value is 0.407, denoting good class separation and confirming that DINOv3 embeddings carry clear class membership information. Importantly, this separability emerges as a result of the full proposed pipeline, including detection refinement and context-aware bounding box expansion, rather than being trivially present in the raw input space.

Hazel Silhouette distribution shifts clearly toward positive values (0.25–0.75), with many samples in the 0.45–0.65 range. This signifies high intra-cluster sample similarity, strong separation from dandelion cluster, and consistent, stable embedding representations. In practice, DINOv3 effectively reflects hazel morphological features in feature space (Figure 21—hazel class—upper section)

Dandelion shows a broader Silhouette distribution: from ~−0.15 to 0.6. Interpretation includes most samples with positive values (properly clustered), some near 0 or negative indicating greater morphological diversity or weaker separation for certain samples, and alignment with biological observation of more visually varied dandelion pollen than hazel. Even so, the distribution leans positive overall (Figure 21, dandelion class—lower section).

Despite the observed class separability, the embedding distributions are not perfectly compact and include borderline samples. Therefore, an explicit decision mechanism is required to define a stable class boundary. In the proposed pipeline this role is fulfilled by the logistic regression classifier, which transforms the geometric separation in the DINOv3 embedding space into an interpretable decision rule.

**Figure 22:**
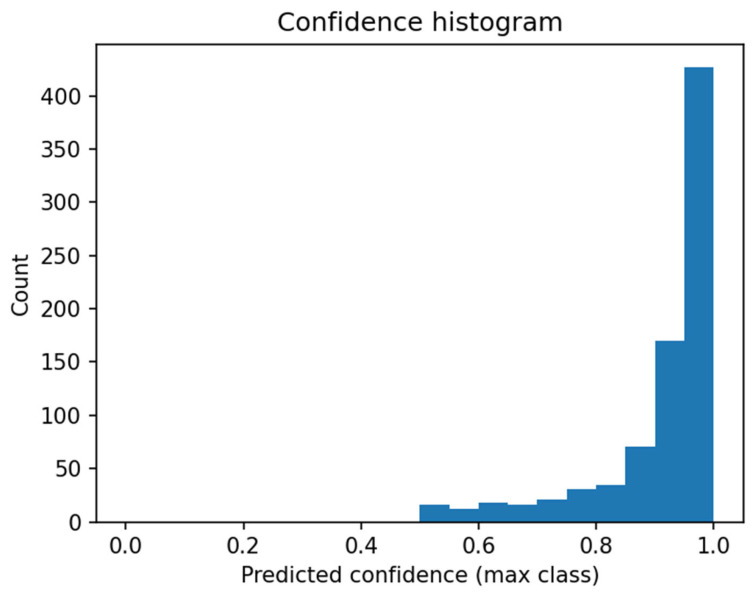
Histogram of prediction confidence for the logistic classifier (DINOv3 embeddings).

The histogram (Figure 22) shows the distribution of maximum prediction confidence obtained by the logistic regression classifier for all test samples. Confidence here refers to the highest class probability returned by the model (softmax).

High Confidence Dominance (0.9–1.0):

The most prominent feature is the strong concentration of predictions in the 0.9–1.0 range. This signifies high separability of DINOv3 embeddings, strong classifier confidence, and absence of difficult cases where confidence would drop to uncertain levels (0.5–0.7). In practice, the model rarely hesitates between classes, receives highly informative embeddings, and unambiguously assigns samples to classes.

No Low-Confidence Predictions (0.0–0.5):

The histogram contains no samples with confidence < 0.5, a highly desirable classifier trait. No random or near-random predictions occur, the model avoids low-confidence decisions, and all decisions remain unambiguous and stable. This rarity in embedding classification confirms excellent classifier fit to the feature space.

Few Intermediate Cases (0.6–0.8):

A few samples achieved confidence 0.6–0.8, representing morphologically challenging intermediate cases such as partially damaged, blurry, or distorted objects, yet the model classifies them correctly with minimal errors. This aligns with the confusion matrix, where errors mainly involved background rejection rather than class confusion.

Logistic Regression Sufficiency:

Logistic regression lacks inherent nonlinearity modeling and relies on linear space partitioning. Such high confidence concentration proves that DINOv3 embeddings are linearly separable, more complex classifiers (SVM, MLP, transformer) would yield no significant improvement, and the YOLO-DINOv3-logistic regression pipeline remains architecturally coherent.

**Figure 23:**
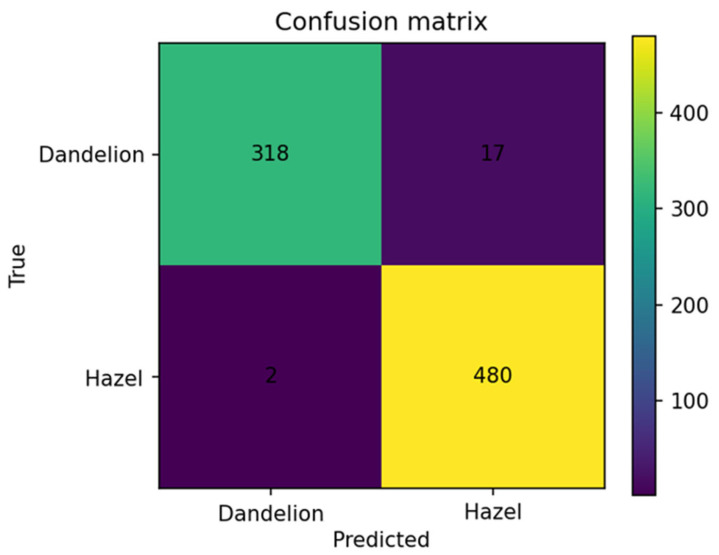
Confusion matrix of the logistic classifier operating on DINOv3 embeddings. The model achieves very high performance, correctly classifying the vast majority of dandelion and hazel samples (accuracy = 98.1%).

The confusion matrix (Figure 23) presents true and false detections by the model of the respective classes:

Dandelion Class:

There are 318 samples correctly classified as dandelion, 17 samples erroneously classified as hazel. This yields Recall(dandelion) = 318/(318 + 17) = 94.9% and Precision(dandelion) = 318/(318 + 2) = 99.4%. The model almost never classifies hazel as dandelion, errors mainly involve morphologically difficult or borderline samples, and dandelion embeddings show good separability but greater diversity (also visible in the Silhouette histogram).

Hazel Class:

There are 480 samples correctly classified, and 2 erroneously classified as dandelion. Results give Recall(hazel) = 480/(480 + 2) = 99.6% and Precision(hazel) = 480/(480 + 17) = 96.6%. Hazel class is very well represented in embedding space, and errors are minimal with nearly all predictions correct, aligning with Silhouette results showing a more compact cluster than dandelion

Overall Classification Quality:

From the matrix, Accuracy = (318 + 480)/(318 + 17 + 2 + 480) = 98.1%

The model makes only 19 errors out of 817 samples, corresponding to very high classification quality for such a small and challenging dataset.

**Figure 24:**
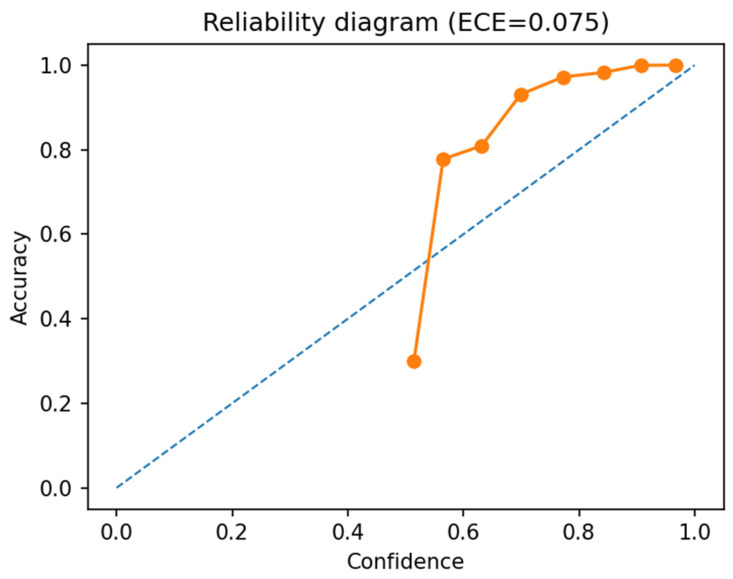
Reliability diagram of the logistic regression-based classifier (ECE = 0.075).

The reliability diagram [43,44] (Figure 24) and low Expected Calibration Error (ECE = 0.075) indicate that the logistic regression-based classifier is well-calibrated and predicts labels with reliable confidence levels. In the dominant high-confidence range, accuracy nearly matches confidence values, evidencing no overconfidence and excellent DINOv3 embedding quality, while minor deviations in the mid-confidence range (0.5–0.7) arise from few difficult cases without impacting overall stability. Ultimately, the classifier exhibits high prediction reliability, essential for automatic microscopic pollen analysis in production settings.

## 3. Results

### 3.1. Ablation: IoU-NMS Threshold

The value of 0.65 represents a compromise between a threshold that is too high, which leaves many overlapping boxes and increases the number of duplicates, and a threshold that is too low, which aggressively reduces boxes at the cost of the risk of losing distinct objects located close to each other.

The threshold value was determined experimentally through a systematic evaluation of different IoU settings. Experimental analysis showed that within the IoU range of 0.30–0.65 the detection recall remains practically unchanged (Table 2). This observation indicates that the NMS threshold does not significantly affect the sensitivity of the detector in this range. Instead, its primary impact concerns the control of redundant detections rather than the ability to localize objects.

Consequently, the choice of IoU = 0.65 ensures effective suppression of duplicate bounding boxes while preserving a stable and high recall, which is particularly important in microscopy images characterized by dense object distributions.

Experiments conducted showed that for the IoU range = 0.30–0.65, the obtained recall remains practically identical, whereas thresholds (≥0.80) lead to an increased number of duplicate detections (Table 2).

### 3.2. Ablation: Bounding Box Expansion

In the second stage, crops were prepared. Each bounding box detected by YOLO is then expanded by 12% of its width and 12% of its height. This procedure allows for preserving a wider context around the object, which proved crucial for the quality of feature extraction by DINOv3.

Empirical testing of bounding box padding demonstrated a clear trade-off between contextual information and background noise. While insufficient padding hindered classification by omitting edge details (Table 3), values exceeding 0.12 introduced noise that degraded model accuracy. The optimal balance was found at a 0.12 expansion, which provided the most stable feature embeddings for the DINOv3 architecture while maintaining a high signal-to-noise ratio.

### 3.3. Graphical Results

**Figure 25:**
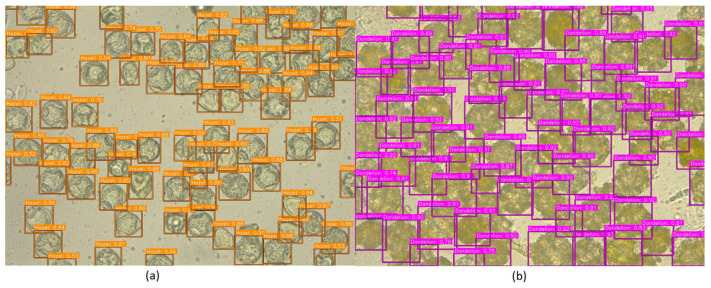
Visual output of presented model (**a**) detected hazel grains (**b**) detected dandelion grains.

The final output of the model gives detection bounding boxes (Figure 25), labels and confidence of prediction.

### 3.4. Comparison with Shallow and Deep Learning Baselines

The proposed classification stage was evaluated against one shallow baseline and one conventional deep learning baseline under a controlled evaluation protocol. The detector (YOLOv12m) and the ROI generation procedure were kept unchanged across all compared variants, and all models were evaluated on identical ROI crops extracted from bounding boxes using the same context-aware padding strategy. This experimental design ensures that any observed differences can be attributed to the classification architecture and feature representation rather than to discrepancies in ROI generation.

For the shallow baseline, HOG descriptors were extracted from ROI crops and classified using an SVM. For the deep learning baseline, an ImageNet-pretrained ResNet model was fine-tuned end-to-end on the same ROI crops for the same binary classification task. Table 4 reports the results on the same test set as that used in the ablation study.

**Figure 26:**
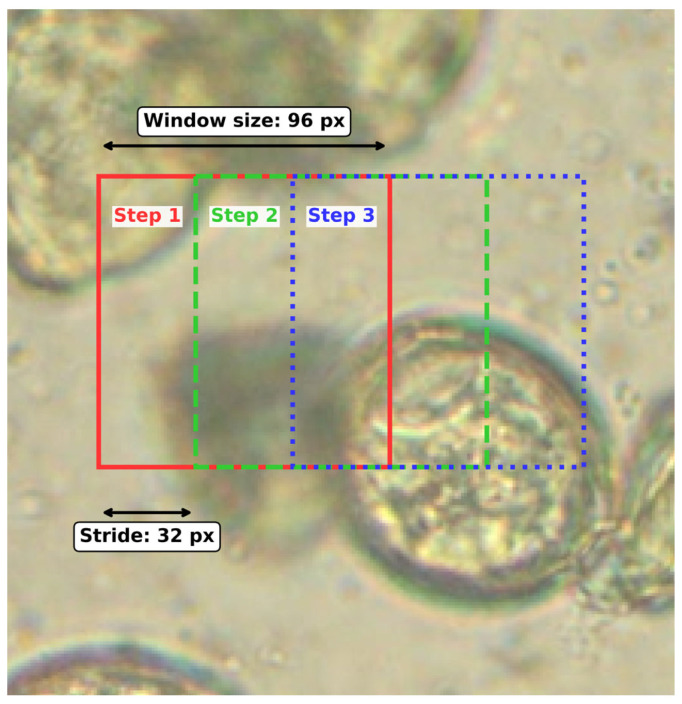
Illustration of the sliding-window scanning strategy employed in the classical HOG-SVM baseline.

As a shallow object-detection baseline, we implemented a classical HOG–SVM sliding-window detector operating directly on the full microscopy images. The model was trained on window crops extracted from annotated bounding boxes (positive samples) and from background regions without overlap with ground truth (negative samples). During inference, each image was scanned using a sliding window with stride 32 pixels and the window size was selected to approximate the characteristic scale of pollen grains. Because pollen objects exhibit variability in size, multiple window configurations were evaluated (multi-scale scanning) instead of a single fixed crop. One representative configuration (crop size 96 × 96 pixels, stride 32 pixels) is illustrated in Figure 26.

**Figure 27:**
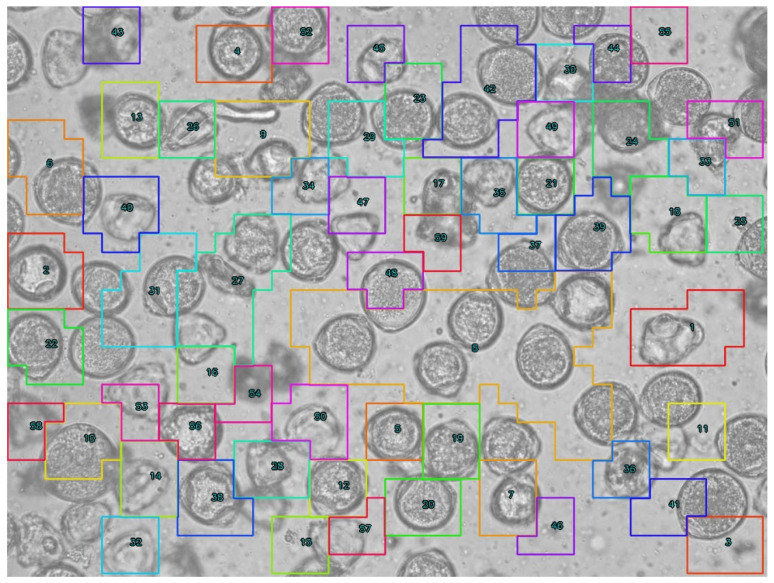
Detection results obtained using the shallow HOG-SVM baseline, where different colors distinguish adjected regions and numbers denote individual detected objects.

Each window was represented using HOG descriptors and classified with an SVM. Candidate detections were retained when the SVM decision score exceeded a threshold of 0.75. Owing to the dense scanning strategy, many neighboring windows generated highly overlapping detections. Consequently, Non-Maximum Suppression was applied: detections with overlap greater than 50% (i.e., IoU > 0.5) were merged by retaining the bounding box with the highest score. Despite this post-processing, the method remains computationally inefficient because it requires evaluating a large number of windows per image and, as illustrated in Figure 27, produces substantially lower localization and classification accuracy compared with the deep learning-based detection pipeline.

**Figure 28:**
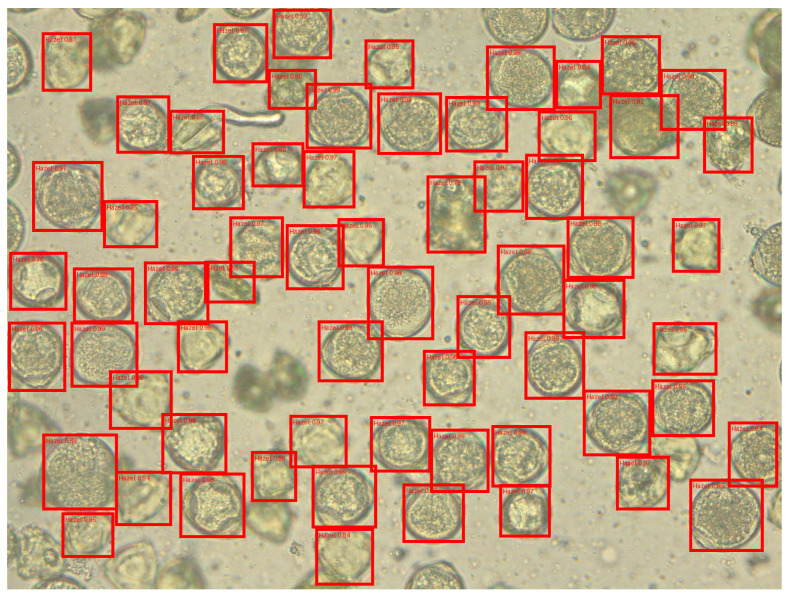
Detection results obtained using the fine-tuned ResNet-50-FPN model.

To evaluate the performance of standard convolutional neural networks, a deep learning pipeline based on ResNet-50-FPN was implemented. Transfer learning was utilized by initializing the network with weights pre-trained on the MS COCO dataset, followed by fine-tuning on the annotated pollen microscopy imagery. The feature Pyramid Network architecture was selected to leverage multi-scale feature maps. While this approach demonstrates superior performance compared to the classical HOG-SVM baseline, it remains outperformed by the modular framework proposed in this study. The corresponding detection results are presented in Figure 28.

## 4. Discussion

### 4.1. Results Interpretation in Literature Context

The high effectiveness of the approach stems primarily from its novel modular architecture, which separates ROI detection, feature extraction, and classification decision-making. YOLOv12m serves as the ROI generator, DINOv3 produces 1024D embeddings, and logistic regression makes the final decision. This division limits error propagation and enables independent stabilization of the stages most sensitive to microscopic data variability (especially ROI generation).

A second key factor is the use of DINOv3 ConvNeXt-B as a feature extractor pretrained on a very large and diverse dataset, which reduces the need for training from scratch on annotation-scarce data and enhances representation stability. Consequently, the feature space is well-structured, allowing the decision boundary to be largely linear. The choice of logistic regression is not arbitrary but a deliberate capacity constraint: with highly separable embeddings, more complex classifiers offer no substantial gains, while this simple method minimizes overfitting risk on small datasets.

While the current model demonstrates high accuracy, it is important to note that this study serves as a proof of concept developed using single-taxon slide preparations. These controlled conditions allowed for the isolation of morphological features but do not yet account for the complex matrices found in commercial honey.

### 4.2. Ablation Findings

In this work, two controlled analyses of detection-to-embedding interface parameters were performed, which directly influence the quality of the entire pipeline. The first involved selection of the IoU threshold in NMS, where a value of 0.65 was chosen as a compromise that reduces duplicate detections without significant loss of sensitivity; this is particularly important for numerous small objects in microscopic images.

The second analysis examined contextual expansion of the bounding box by 12%. It was shown that standard bounding boxes may truncate essential morphological pollen features, while ROI expansion improves embedding separability and final classification effectiveness. This serves as an ablation study demonstrating the following tradeoff: overly tight framing removes information, while excessive background addition introduces noise.

The results of these analyses suggest that the key advantage does not stem solely from architecture selection, but from systematic standardization of ROI generation, ensuring repeatable and comparable inputs to the feature extractor and mitigating errors arising at stage boundaries.

### 4.3. Limitations and Practical Applications

Preparing training datasets for artificial intelligence-based models requires significant effort. Key aspects include acquiring pollen-producing plants during the appropriate phenological period, which may limit the availability of representative biological material under seasonal conditions. Additionally, optimizing the preparation of microscopic slides in terms of pollen density is crucial to avoid excessive overlapping of grains or too few grains per field of view, which could affect the quality and accuracy of the analysis. Manual annotation of each pollen grain remains a time-consuming and labor-intensive process, posing a significant limitation to the rapid development and scalability of automated methods. For carefully prepared slides, semi-automatic or fully automatic methods dedicated to initial annotation can be applied, substantially reducing the time required for training data preparation.

The presence of pollen grains in various developmental stages—such as immature, mature, or damaged grains—introduces additional variability into the dataset, negatively impacting both manual annotation processes and the effectiveness of artificial intelligence classification models. Morphological differences between individual developmental stages can be difficult to interpret unambiguously even for experienced experts, increasing the risk of human-in-the-loop errors and causing heterogeneity in training data. As a result, models trained on such material may exhibit reduced precision and greater susceptibility to misclassifications.

To mitigate this issue, preliminary data selection or filtering stages can be introduced, along with expanding the training set to include samples representing the full spectrum of pollen conditions, which can enhance the model’s generalization ability. Furthermore, applying data augmentation techniques and advanced transfer learning methods can support model adaptation to pollen morphological diversity. Integrating expert knowledge into the annotation and result evaluation processes will be essential for maintaining high quality and reliability of automated analysis.

Beyond the challenges of data acquisition and annotation, a critical limitation of the current study is its taxonomic scale. This research utilized only two to demonstrate the fundamental performance of the YOLOv12m-DINOv3 pipeline. While the system achieved exceptionally high accuracy (98.1%) under these conditions, such results are expected in binary classification and may not directly translate to multi-class settings involving dozens of taxa.

In real-world melissopalynology, the presence of morphologically similar pollen types from the same botanical family significantly increases the risk of inter-class confusion. As the number of taxa grows, the feature space becomes more crowded, potentially reducing the linear separability currently observed in our DINOv3 embeddings. Therefore, the high performance reported here must be interpreted as a validation of the architectural framework rather than a final measure of its taxonomic scalability.

The practical applications of the developed system primarily focus on combating honey adulteration, which poses a serious challenge to the local market, negatively impacting both product quality and consumer trust. Adulteration, involving the addition of cheaper substitutes or the import of low-quality honey from non-EU countries, leads to destabilization of the domestic market and generates significant financial difficulties for beekeepers. Automating honey pollen analysis can contribute to streamlining quality control processes and standardizing research procedures, while also reducing costs and the risk of errors associated with manual diagnostics.

Once validated against heterogeneous commercial samples, the implementation of such a method could eventually offer a cost-effective tool for honey adulteration. However, routine application for adulteration detection remains contingent upon further testing against real-world samples containing damaged grains and complex debris.

Furthermore, the continuous development of a database containing various pollen types on which the model is trained will enable increased classification effectiveness and precision, ultimately translating into improved quality and reliability of automated methods for honey quality analysis.

A significant limitation of the current dataset is the absence of non-pollen particulates such as wax fragments, yeast cells, and soot—which are ubiquitous in raw honey samples. In real world honey matrices, these artifacts can trigger false positives in automated systems.

### 4.4. Error Analysis

Analysis of the error patterns indicates that the weaker detection quality for hazel is not primarily due to confusing the hazel class with dandelion, but rather to a higher rate of discarding hazel objects as background (false negatives, FN). In the detector’s confusion matrix, there are no mistakes where hazel is classified as dandelion; instead, a substantial portion of hazel instances are misclassified as background (119 cases).

Specifically, for hazel, we obtained 425 correct detections and 119 misses, and for dandelion, 334 correct detections and 49 misses. This behavior mirrors the trend reported by Shi X. [45], who demonstrated that for YOLO-based pollen detection systems the dominant source of performance degradation is not inter-class confusion but missed detections and background-related errors. Similar conclusions were drawn in their study, where recall degradation was attributed primarily to challenging object appearances rather than incorrect class assignment.

This tendency is further reflected in our F1–Confidence analysis (Figure 6), where recall remains stable across a wide confidence range, while most errors originate from background suppression rather than misclassification. Additionally, false positives predominantly arise from background structures resembling hazel grains, which explains the lower precision observed for this class. Background is thus much more frequently classified as hazel than as dandelion.

Importantly, the classifier performance on learned embeddings remains very high, which further suggests that the fundamental limitation for hazel lies upstream of the classification stage, rather than the separability of representations in the embedding space.

### 4.5. Comparison with Related Works and Ablation Strategies

To better position the proposed approach with respect to existing pollen detection pipelines, Table 5 summarizes the scope and focus of ablation analyses reported in related works. The comparison emphasizes differences in pipeline structure and the aspects subjected to systematic evaluation, providing context for the design choices adopted in this study.

### 4.6. Future Studies

Future work should focus on developing a standardized procedure for preparing microscopic slides and ensuring optimal pollen grain density and distribution on the slide, which will minimize artifacts such as grain overlapping or empty areas in the field of view. A key element will involve systematically investigating the impact of microscope parameters—including optical magnification, lighting type (transmissive or fluorescent), and contrast—on imaging quality and AI model classification accuracy, enabling the selection of universal settings independent of specific laboratory equipment.

The next priority will be the development and validation of automatic or semi-automatic pollen grain annotation methods, utilizing image segmentation algorithms (e.g., U-Net) and zero-shot classification models, which will significantly shorten training set preparation time. Systematic analysis of annotation quality’s influence on model metrics (precision, recall, F1 score) will allow determination of minimum requirements for input data.

Experiments will be conducted to determine the minimum number of pollen samples required to achieve models with satisfactory performance (>90% accuracy), employing data augmentation techniques (rotations, deformations, lighting changes) and active learning methods. These tests will also include analysis of learning curves as a function of training set size.

As a concrete next step toward practical implementation, we will focus on expanding the pollen database to include more common nectar-producing species found in Central European honeys. This expansion will be coupled with testing on mixed-sample images and authentic honey preparations, moving away from single-taxon slides. This will allow us to evaluate the model’s robustness in ‘Melissopalynology-in-the-wild’ scenarios, where the system must differentiate target pollen from environmental debris and handle the high overlap characteristic of dense honey sediments.

The effectiveness of transfer learning will be examined by training models on images from one microscope and testing them on data from various optical systems, evaluating performance degradation and fine-tuning efficiency. Expanding the database with additional pollen-producing plant species will enable creation of a universal model recognizing dominant pollen sources in multifloral honeys.

The ultimate goal will be to develop an integrated pipeline for automatic honey pollen analysis, deployable in quality control laboratories and apiaries, featuring an interface for rapid sample diagnostics under field conditions.

Because honey variety, corbicular pollen and bee bread composition analysis relies on the relative percentage contribution of individual pollen types, the system must be able to recognize all pollen taxa that may occur in a given honey product. Therefore, further development of the proposed system will also focus on extending the model to multi-class recognition, supported by unsupervised representation learning such as autoencoding to capture discriminative morphological features and improve robustness to intra-class variability.

Crucially, subsequent research must focus on cross-validation using commercial honey samples. This will involve testing the model’s ability to perform “Melissopalynology-in-the-wild”, where the system must differentiate target pollen from environmental debris.

## 5. Conclusions

This approach enabled the introduction of proprietary modifications to the detection stage, particularly contextual expansion of detection frames and optimization of the IoU parameter in the Non-Maximum Suppression algorithm, which were not considered in the reference work. The conducted ablation study analysis showed that moderate expansion of detection frames by approximately 12% leads to an increase in the detector’s average recall from 0.937 to 0.944 (≈+0.7 p.p.), while maintaining stable precision.

Importantly, this effect also translates into improved feature representation quality in the subsequent processing stage. For expanded frames, an increase in DINOv3 embedding classification accuracy from 0.990 to 0.997 (≈+0.7 p.p.) was observed, along with a clear improvement in class separability in the feature space, measured by the Silhouette coefficient (average value ≈ 0.41). For larger expansion values (≥16%), a decrease in classification accuracy was noted, confirming the existence of a trade-off between context and introduced background noise.

Additionally, experimental optimization of the IoU parameter in the NMS procedure demonstrated that IoU = 0.65 represents a beneficial compromise between reducing duplicate detections and maintaining high sensitivity, compared to standard settings. The combination of both modifications resulted in more stable predictions and more uniform geometry of the embedding space.

The use of an independent feature extractor and a simple linear classifier additionally enabled direct analysis of the feature space structure, constituting a significant extension compared to approaches based solely on detection metrics. The proposed pipeline demonstrates potential for better domain generalization, particularly under conditions of a small number of classes and high morphological variability of microscopic objects.

The obtained results indicate that the proposed modifications to the detection stage lead to genuine improvement in the quality of the entire pipeline, rather than merely local optimization of a single metric. The increase in detector recall following contextual expansion of detection frames demonstrates better coverage of the full pollen grain structure, which is crucial in microscopic images where key morphological features are often located at object boundaries.

The simultaneous improvement in embedding classification accuracy and increase in class separability in the feature space indicate that the input signal provided to the DINOv3 extractor is more informative and consistent. This means that frame expansion does not introduce random context but delivers useful information supporting class discrimination, confirming the effectiveness of the proposed approach.

The analysis conducted as a function of the frame expansion parameter revealed a clear optimum. For small expansion values, insufficient representation of morphological context was observed, while for larger values, a decline in classification quality occurred due to excessive background noise introduction. The fact that a maximum occurs for moderate expansion (≈12%) confirms that the proposed modification is systematic rather than random.

Analogously, selecting the IoU parameter in the Non-Maximum Suppression algorithm at the level of 0.65 allowed for reducing the number of duplicate detections while maintaining high sensitivity. This indicates that standard detector settings are not optimal for high-density microscopic images, and their adaptation to domain specifics leads to measurable benefits.

Overall, the achieved results confirm that the proposed detection stage extensions improve the stability and consistency of feature representations, translating into higher final classification effectiveness. The method exhibits robustness to morphological variability and potential for generalization to other microscopic datasets, making it a useful extension of standard YOLO-based pipelines.

The conducted research provides a robust proof-of-concept demonstrating that artificial intelligence-based methods are an effective tool for pollen recognition under controlled laboratory conditions. The results obtained remain consistent with the high effectiveness of YOLO models observed in the literature; however, our proposed decoupled pipeline using YOLOv12m as an ROI [46,47] generator and DINOv3 for feature extraction offers a significant advancement in morphological representation.

This study successfully demonstrated that a moderate contextual expansion of detection frames (≈12%) increases the detector’s average recall and improves class separability in the feature space. Furthermore, optimizing the IoU parameter to 0.65 addressed the specific challenges of high-density microscopic imaging. These modifications lead to a genuine improvement in the quality of the pipeline, providing more informative input signals for classification than standard end-to-end models.

However, it is important to explicitly frame these findings within the scope of the current dataset. As the model was developed using single-taxon slide preparations, the high accuracy metrics (up to 0.997) reflect the system’s potential under ideal conditions rather than its immediate readiness for commercial use. The current study does not yet account for the complexities of real-world honey matrices, which include: mixed taxa and inter-species morphological similarities, the presence of botanical debris, wax fragments, and non-pollen particulates, heavy grain overlap and damaged grains typical of commercial honey sediment.

While the proposed method exhibits strong robustness to morphological variability, further validation on heterogeneous, real-world honey samples is required before it can be implemented for routine honey authentication or adulteration detection. The high performance reported here serves as a critical benchmark for the system’s morphological capabilities.

Ultimately, the greatest potential lies in a hybrid approach where AI serves as a valuable complement to, rather than a full replacement for, classical palynological expertise. Future research will focus on bridging the gap between this proof-of-concept and a field-deployable tool by training the model on “noisy” environmental samples and multi-class honey sediments. This will be a necessary step toward the practical automation and standardization of honey quality analysis in apiaries and quality control laboratories.

## Figures and Tables

**Table 1:** Average inference-time characteristics of the proposed pipeline. The feature extraction cost scales with the number of detected regions of interest, which averaged approximately 40 per image.

Pipeline Component	Average Inference Time
YOLOv12m detection	174.74 ms/image
DINOv3 feature extraction	335.91 ms/image
Logistic regression classification	0.56 ms/image

**Table 2:** Influence of the IoU threshold in the Non-Maximum Suppression (NMS) algorithm on detection recall and duplicate detections.

IoU NMS Threshold	Average Detection Recall	Average Duplicates per Ground-Truth Object
0.3	0.94	1.1726
0.5	0.94	1.1726
0.65	0.94	1.1726
0.8	0.94	1.1738

**Table 3:** Effect of context-aware bounding box padding on classification accuracy for correctly matched detections.

Classification Accuracy on Matched Objects	Bounding Box Padding Ratio
0.0	0.987
0.8	0.997
0.12	0.997
0.16	0.996

All ablation results are reported on a fixed test set. Each value represents the average over all test images.

**Table 4:** ROI-level baseline comparison on the fixed test set (correctly matched detections). Metrics: overall accuracy and per-class precision/recall.

Method	Type	Accuracy	Precision (Dandelion)	Recall (Dandelion)	Precision (Hazel)	Recall (Hazel)
HOG + SVM	shallow	0.678	0.660	0.640	0.690	0.610
ResNet (fine-tuned)	deep	0.711	0.822	0.859	0.600	0.744
DINOv3 + Logistic regression (proposed)	deep (modular)	0.981	0.994	0.949	0.966	0.996

**Table 5:** Comparison of ablation analysis scope in detection pipelines.

Method	Pipeline Organization	Explicit Embedding Stage	Detection–Classification Interface Ablation	Annotation-Level Ablation	Feature-Level Ablation
This work (YOLOv12m + DINOv3 + logistic regression)	modular	yes, DINOv3	yes, ROI expansion, NMS IoU tuning	no	no
Jofre et al., 2025 [16]	end-to-end, YOLO detector	not reported	no	no	no
Gimenez et al., 2024 [18]	end-to-end, YOLO detector	not reported	no	yes, all-0taxon, all-5taxa, 1taxon-taxon	no
Olsson et al., 2021 [14]	partially modular, ROI extraction + CNN classifier	no	no	no	yes, CNN architecture comparison
Kubera et al., 2022 [19]	end-to-end, YOLO detector	no	no	no	no
Shi et al., 2026 [45]	end-to-end detection framework	not reported	no	no	yes, handcrafted morphological features
J. Wu et al., 2020 [46]	partially modular, detector + explicit ROI cropping + classifier	yes	yes, ROI Pooling, Crop + resize, ROI expansion	no	yes, context fusion variants
Zhang et al., 2020 [47]	end-to-end	no	partial, ROI Context fusion	no	yes, attention design

## Data Availability

The data will be made available upon reasonable request from the corresponding author.
